# The Malady of Microbes and Maggots

**DOI:** 10.4269/ajtmh.21-0641

**Published:** 2021-08-30

**Authors:** Karthikeyan Kaliaperumal, M. Prarthana

**Affiliations:** ^1^ Department of Dermatology, Venereology, and Leprosy, Sri Manakula Vinayakar Medical College and Hospital, Puducherry, India;; ^2^ Department of Dermatology, Venereology, Leprosy, Sri Manakula Vinayakar Medical College and Hospital, Puducherry, India

Leprologists routinely encounter leprosy cases with various presentations during daily practice. Patients continue to experience leprosy-related stigma and prejudice that prevent them from seeking care despite the intensified eradication program. For example, one patient presented to our clinic with a trophic ulcer infested with maggots. She had a case of burnt-out Hansen’s disease for 40 years resulting in deformity of both feet and trophic changes of her left foot. Embarrassment and the unbearable odor prevented her from seeking health care during the early stage.

This patient served as a reminder of another patient encountered 20 years previously by the first author with a similar presentation. He narrated his experience in his own words. She was a 50-year-old woman with a huge bandage rolled around her right foot. During that time, we managed cases of maggot infestation frequently, and the familiar smell indicated what was underneath her bandage. She walked with difficulty to the examination table and before we could act, she removed the rolled cloth on her foot. This resulted in feelings of extreme nausea and people trying to leave the room. The cloth bandage was caked with pus and serum. Clumps and clumps of maggots fell to the floor and squirmed around, causing anyone who remained in the room to leave. The woman sat there calmly amid the chaos; her eyes were filled with helplessness. I was assigned the role of taking care of her throughout her stay in the hospital.

My days began and ended with removing the maggots because I was assigned to change her bandages twice per day. I felt queasy, but I tried to not show it on my face. While I slept, I would dream of maggots crawling all over me. The smell of turpentine liniment emanated from me. I felt nothing but pity for her, although she endured everything with a serene face. As days passed, we developed a rapport and she talked to me about the initial days of her illness and how she was abandoned by her own family members. She became more cheerful as her condition improved during her 2-month stay. She underwent multiple wound debridements and tissue graft surgery for her nonhealing ulcer and concomitant physiotherapy. On the day of her discharge, I was happy to see her walk with a smile on her face, and I was again reminded of why doctors are considered healers. We cured her discomfort and pain and also gave her the confidence of knowing that she could start a new life.

Presently, at our clinic, there is another woman with a helpless look on her face waiting for a miracle cure. It seems that the stigma associated with Hansen’s disease has not changed much over the years. Ostracization and cultural beliefs combined with poverty drive the fury of maggots thriving on her dead tissue as much as the leprosy-induced trophic ulcer. Although the occurrence of such cases has statistically declined, it still persists. Now, it was his turn to instruct the residents to perform wound care and management with the utmost hope that they will not encounter such cases in the future.

Although multitargeted therapy is recommended by the national leprosy eradication program, the presence of such cases proves the continuous lack of awareness and ability to access preventive and early health care. This indicates the need for social therapy that focuses on re-engineering the psychosocial and public health aspects along with multidrug therapy. Generations of individuals have witnessed the gruesome effects of leprosy on the underprivileged population. With every case of maggots infecting a trophic ulcer, We hope a day will come when the malady of microbes and maggots is only a story to tell and not a case to treat.

